# Trends in the diversity of board-certified pathologists in the United States

**DOI:** 10.1016/j.acpath.2026.100250

**Published:** 2026-05-13

**Authors:** Aanand A. Patel, Bitania Wondimu, Emily Glynn, Nicole R. Jackson, Kareem Hosny

**Affiliations:** Department of Laboratory Medicine and Pathology, University of Washington, Seattle, WA, USA

**Keywords:** Diversity, Equity, Ethnicity, Gender, Pathology, Race, Representation, Workforce

## Abstract

Medicine has historically excluded several marginalized groups, including but not limited to women and people of color. A lack of diversity is an injustice to those excluded from the field and adversely affects the patients we serve. We obtained de-identified demographic data from the American Board of Pathology to examine the diversity of the 39,124 US pathologists certified from its founding through 2024. We focus on pathologists certified since 2006, when continuing certification was introduced and data are most complete. Female representation has steadily increased, with 53% of pathologists certified since 2006 identifying as female compared to 27% of pathologists certified earlier. Nonbinary individuals represent 0.04% of active pathologists. Among active pathologists certified in 2006 or later, 89% identify as White or Asian, and 92% identify as non-Hispanic/Latino/Spanish origin. We observed much lower female representation in clinical pathology only (39%) compared to anatomic only (52%) and anatomic and clinical (54%). Representation varies across subspecialties, with the lowest female representation in clinical informatics (25%). This work demonstrates a substantial improvement in female representation among recently certified pathologists, but the overall proportion of women with active certification remains under 50%, and women are underrepresented even among recently certified pathologists in some subspecialties. Several racial and ethnic groups (American Indian or Alaska Native, Black or African American, and Hispanic/Latino) remain severely underrepresented. This work highlights the continued need to recruit and retain a diverse workforce and ensure that all pathologists have equitable access to the full range of career trajectories in our field.

## Introduction

Medicine has historically excluded several marginalized groups, including but not limited to women and people of color (POC). A lack of diversity among physicians is not only an injustice to those excluded from the field, but also adversely affects the patients we serve. Evidence from across disciplines demonstrates that diverse teams perform better; healthcare providers from underrepresented minority groups disproportionately care for underserved patient populations, and diversity of healthcare providers improves patient outcomes.^1^, ^2^, ^3^

Several studies across a variety of medical specialties have characterized the diversity of the physician workforce.^4^, ^5^, ^6^, ^7^, ^8^, ^9^, ^10^, ^11^, ^12^, ^13^ These data show some improvements in diversity that are most pronounced among medical students and residents, particularly with respect to gender as approximately 50% of medical students are now women.^4^ In contrast, the diversity of practicing physicians has lagged behind. Data from the US Physician Workforce Data Dashboard show that in 2023, 38.1% of the active physician workforce was female. The extent of female representation varied widely by specialty, ranging from 6.5% female physicians in orthopedic surgery and 7.7% in sports medicine up to 63.0% in palliative medicine and 66.2% in pediatrics.^6^ Progress with respect to racial and ethnic diversity has been very limited, and minority racial/ethnic groups remain severely underrepresented across medical specialties, often with less than half the expected Black and Hispanic representation based on the US census.^7^, ^8^, ^9^^,^^12^, ^13^, ^14^

The diversity of attending physicians may be expected to automatically improve as more diverse cohorts of medical school and residency programs join the workforce and older, less diverse generations of physicians retire. This projection assumes that physicians of all demographics enter and remain in the workforce and medical specialties at equal rates. However, underrepresented groups may face higher attrition rates throughout and after medical training. The metaphor of a “leaky pipeline” has been used to describe the higher attrition rates for women and POC in medicine and many other science, technology, engineering, and mathematics (STEM) fields due to a variety of factors, including a lack of mentorship, an unwelcoming training or working environment, and experiences with bias and discrimination.^15^, ^16^, ^17^, ^18^, ^19^, ^20^, ^21^, ^22^ Therefore, it is important to not only diversify medical schools and training programs but also ensure that trainees from marginalized backgrounds are equitably hired and retained as physicians across the wide range of career paths.

Recent work has specifically characterized the diversity of pathologists using data extending up to 2018.^5^ These data demonstrate increasing diversity among pathologists, particularly pathology residents, and correlate with similar trends seen to varying degrees in other medical specialties such as medical oncology, dermatology, and orthopedic surgery.^7^^,^^8^^,^^12^ Since 2006, the American Board of Pathology has instituted a requirement of continuing certification, providing a comprehensive dataset of board-certified pathologists actively practicing in the United States. Here, we examined this dataset to describe the current state of the pathologist workforce in the United States, compare the current state to prior data, and identify differences among pathology subspecialties.

## Materials and methods

### Dataset

The American Board of Pathology provided de-identified demographic data for the 39,126 people who have been certified on February 14, 2025, or earlier. All certified pathologists are asked to provide their gender identity at the time of their application to the American Board of Pathology. Demographic questions were expanded on January 13, 2022, to include race and ethnicity. These questions are now asked of all new applicants. Pathologists who were certified before this date are also prompted to answer the demographic questions when they log onto the PATHway website (the certification management system). The data are most complete and up to date for the 11,807 pathologists who were certified in or after 2006, when Continuing Certification was implemented because these pathologists must log into PATHway regularly to maintain their certification. The information provided for each board-certified pathologist included the following.

(1) Gender: Pathologists have always been asked to provide their gender identity. Historically, female and male were the only available options. As of January 13, 2022, the options were expanded to female, male, nonbinary, or prefer not to answer. Gender identity was available for all but 527 pathologists who indicated “prefer not to answer,” and these pathologists were excluded from gender analyses.
(2) Race: Pathologists are asked by the American Board of Pathology to indicate their race with the question, “How would you best describe yourself?” This question was introduced on January 13, 2022. The provided options, following the racial categories historically used by the U.S. Census Bureau, are (a) American Indian or Alaska Native, (b) Asian, (c) Black or African-American, (d) Native Hawaiian or Other Pacific Islander, (e) other (with a text field to specify), or (f) prefer not to answer. More than one race can be selected. Most pathologists certified prior to 2006, when continuing certification was introduced, do not have their racial identity on file. Race data were unavailable for 2144 of the pathologists certified in or after 2006.
(3) Ethnicity: Pathologists are asked, “Are you of Hispanic/Latino/Spanish origin?” with the following options: Yes, No, or prefer not to answer. This question was also introduced on January 13, 2022, and a response is not available for most pathologists certified prior to 2006. Ethnicity data were unavailable for 1703 of the pathologists certified in or after 2006.
(4) Primary certification: The American Board of Pathology certifies pathologists in anatomic pathology (AP) only, clinical pathology (CP) only, or both anatomic and clinical pathology (AP/CP).
(5) Primary certification date. For some pathologists, the primary certification is grouped together with a secondary subspecialty certification (for example, anatomic pathology and neuropathology or AP/NP). In these instances, we considered the date of dual certification as both the primary and secondary certification dates.
(6) Primary certification status (active, reinstated, expired, revoked, or surrendered). We grouped pathologists whose certification was reinstated with the active pathologists. The expired, revoked, and surrendered categories were grouped together as “inactive.” Pathologists with inactive primary certification were excluded, except for the analyses incorporating all board-certified pathologists or specifically comparing active versus inactive certification.
(7) Secondary certification: The American Board of Pathology certifies pathologists in the following subspecialties, with the indicated number of active pathologists certified since 2006: Blood banking/Transfusion medicine (759), Chemical pathology (7), Clinical informatics (301), Cytopathology (2,479), Dermatopathology (876), Forensic pathology (828), Hematopathology (2,698), Medical microbiology (128), Molecular genetic pathology (1,083), Neuropathology (483), and Pediatric pathology (421). The data include the name of each certified subspecialty, the certification date, and the status. Chemical pathology was excluded from our analysis of specific subspecialties because of the limited number of pathologists with available demographic data.
(8) Graduate degree.
(9) Occupation status (active or retired) and physical status (alive or deceased). Pathologists who are retired or deceased were considered inactive and excluded from most analyses, except when specified as including all certified pathologists.

### Comparison data

Demographic data for US medical students and the physician workforce were obtained from the US Physician Workforce Data Dashboard, published by the Association of American Medical Colleges.^6^ Demographic data for the United States population were obtained from the US Census using population estimates for July 1, 2024.^23^

## Results

### Gender diversity

**Fig. 1:**
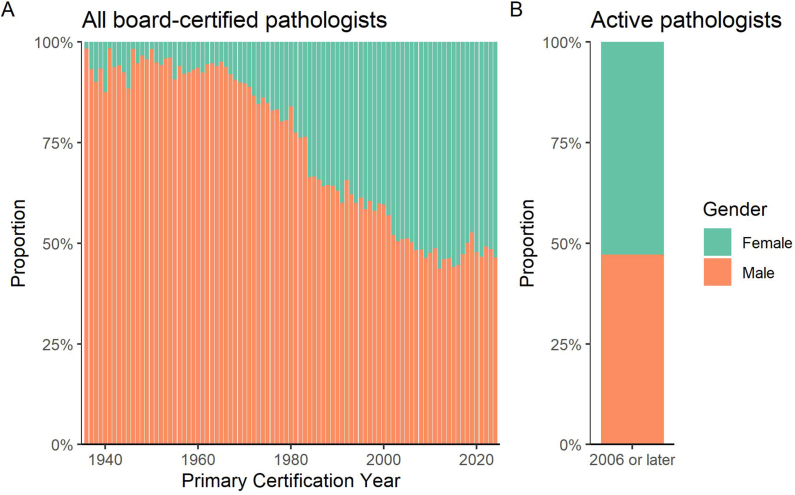
**Gender of board-certified pathologists in the United States.** (A) Gender identity of 37,961 pathologists who have been certified by the American Board of Pathology from inception to 2004 and provided their gender, by year of certification. Female representation has dramatically increased with time, and in the last several years more than 50% of newly certified pathologists identify as female. Individuals who preferred not to provide their gender identity are excluded. Individuals who identify as nonbinary are omitted from this figure because the proportion of pathologists identifying as nonbinary is too small to display (0.04% of all pathologists). (B) Gender identity of 10,997 pathologists with active board certification who were certified after the introduction of continuing certification in 2006.

The American Board of Pathology has, since its inception, collected data on the gender of pathologists at the time of their certification, allowing us to track female representation of newly board-certified pathologists over time. Female representation among all pathologists who have been board-certified in the US has steadily increased (Fig. 1A). This trend follows the steady increase in female representation among matriculants to US medical schools, which increased from 29% in 1980–1981 to 55% in 2024–2025, surpassing 50% for the first time in the 2017–2018 academic year.^6^

While the American Board of Pathology has collected data on gender since its inception, data on race and ethnicity were only introduced recently. Race and ethnicity data are most complete for pathologists certified in or after 2006, when continuing certification was introduced. Since our analyses focus on this more recent cohort, we specifically examined gender representation among active pathologists certified after 2006 (Fig. 1B). Fifty-three percent of pathologists in this cohort are female. All subsequent analyses include only pathologists with active certification from 2006 or later, unless otherwise specified.

We note that the option to indicate a nonbinary gender identity in PATHway was only introduced in 2022, and some pathologists who identify as nonbinary may not have changed their gender selection in PATHway after this introduction. Only 14 active pathologists indicated a nonbinary identity (0.04%), and most were certified in the last 10 years. The proportion of nonbinary pathologists was too small to display clearly and was excluded in Fig. 1A and B. We also excluded nonbinary pathologists from subsequent analyses to avoid the possibility of an individual being identified.

### Racial and ethnic diversity

**Fig. 2:**
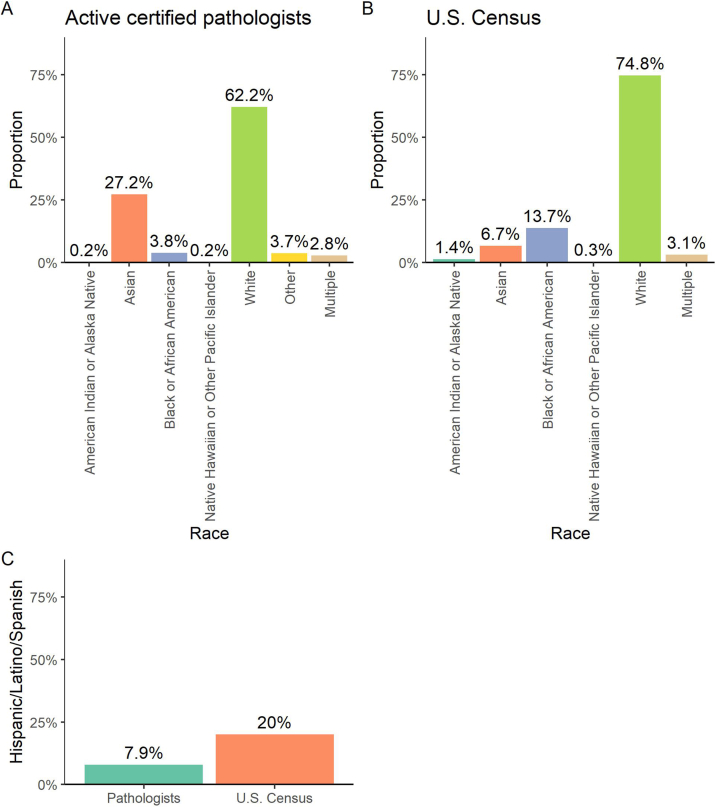
**Race and ethnicity of active pathologists with continuing certification.** (A) Race of 9597 active board-certified pathologists. Pathologists are asked for their racial identity, with the option to select from the US Census categories, select “other” with a free text field to specify, or indicate “prefer not to answer” (excluded from this figure). (B) Race data from the 2025 US Census. The US Census does not provide an “other” option. (C) Ethnicity of 10,035 active board-certified pathologists, compared to 2025 US Census data. Pathologists were asked whether they identify as having Hispanic/Latino/Spanish origin. Those who selected “prefer not to answer” are excluded.

Among active pathologists certified in 2006 or later for whom race data are available, 62% are White, 27% Asian, 4% Black or African American, 0.2% American Indian or Alaska Native, 0.2% Native Hawaiian or Other Pacific Islander, 4% other, and 3% selected multiple racial identities (Fig. 2A). For comparison, the US Census estimates for race in the overall US population are as follows: 75% White, 14% Black, 7% Asian, 1% American Indian or Alaska Native, 0.3% Native Hawaiian or Other Pacific Islander, and 3% multiple (Fig. 2B).^23^ The US Census does not provide an “Other” option, limiting direct comparison between our data and the census data. Eight percent of pathologists identified as Hispanic, Latino, or Spanish origin, excluding those who did not provide a response (Fig. 2C). In comparison, 20% of the US population identifies as Hispanic or Latino.^23^

The US Census racial categories are very broad and do not capture many more specific racial and ethnic identities. To explore which identities are not captured in these data, we examined and aggregated the 464 “other” responses that pathologists included in free text. Commonly provided responses included Middle Eastern and North African (156), South Asian (95), and Hispanic/Latin American/Caribbean (88) identities, with many specifying a particular nationality or ethnic group within these categories. Pathologists with multiple racial identities commonly listed their race as “multiracial,” “biracial,” “mestizo/a,” or “mixed,” and/or specified their identities in the “other” field.

**Fig. 3:**
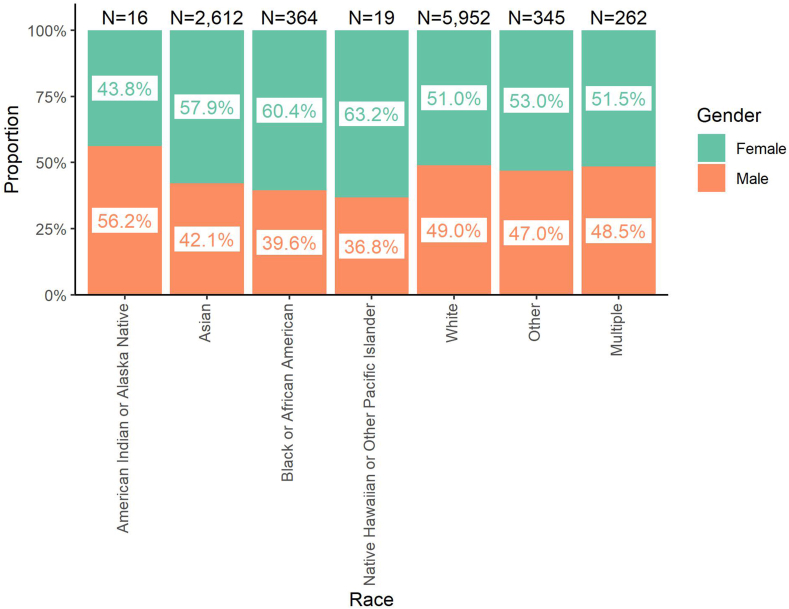
**Intersection of race and gender.** The proportion of pathologists identifying as female or male is shown for each racial category, demonstrating a lower proportion of males in the Asian and Black or African American groups. Data are shown only for pathologists with active continuing certification. N values shown above each bar indicate the sample size of active pathologists in each race who indicated their gender as female or male.

To explore the intersection between race and gender, we evaluated the proportion of active pathologists in each racial category who identify as female vs male (Fig. 3). We found that gender representation varies according to racial group, with a lower proportion of male pathologists among those identifying as Asian or Black or African American.

### Gender and race by subspecialty

**Fig. 4:**
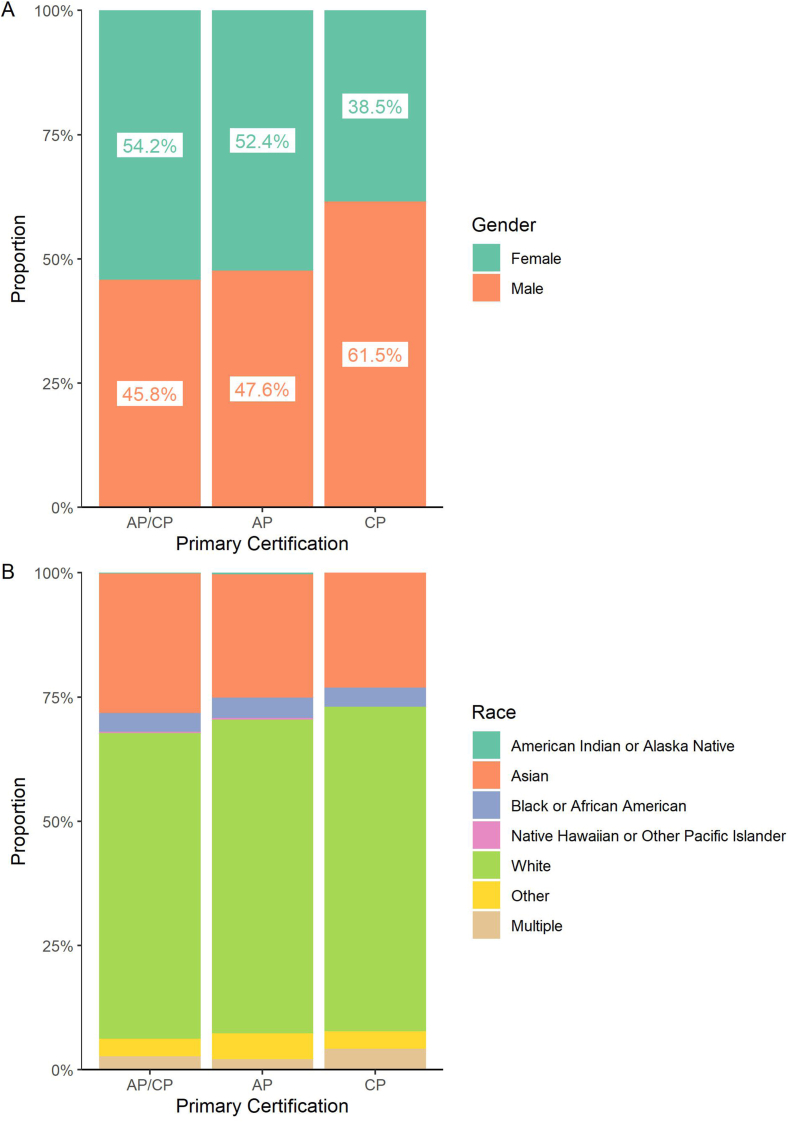
**Gender and race of active pathologists according to certification in AP/CP, AP only, or CP only.** (A) Gender of 10,786 active pathologists by primary certification (8620 AP/CP, 1482 AP only, and 684 CP only). Female representation is markedly lower in CP only compared to AP/CP or AP only. (B) Race of 9423 active pathologists by primary certification (7549 AP/CP, 1280 AP only, 594 CP only). AP, anatomic pathology; CP, clinical pathology.

**Fig. 5:**
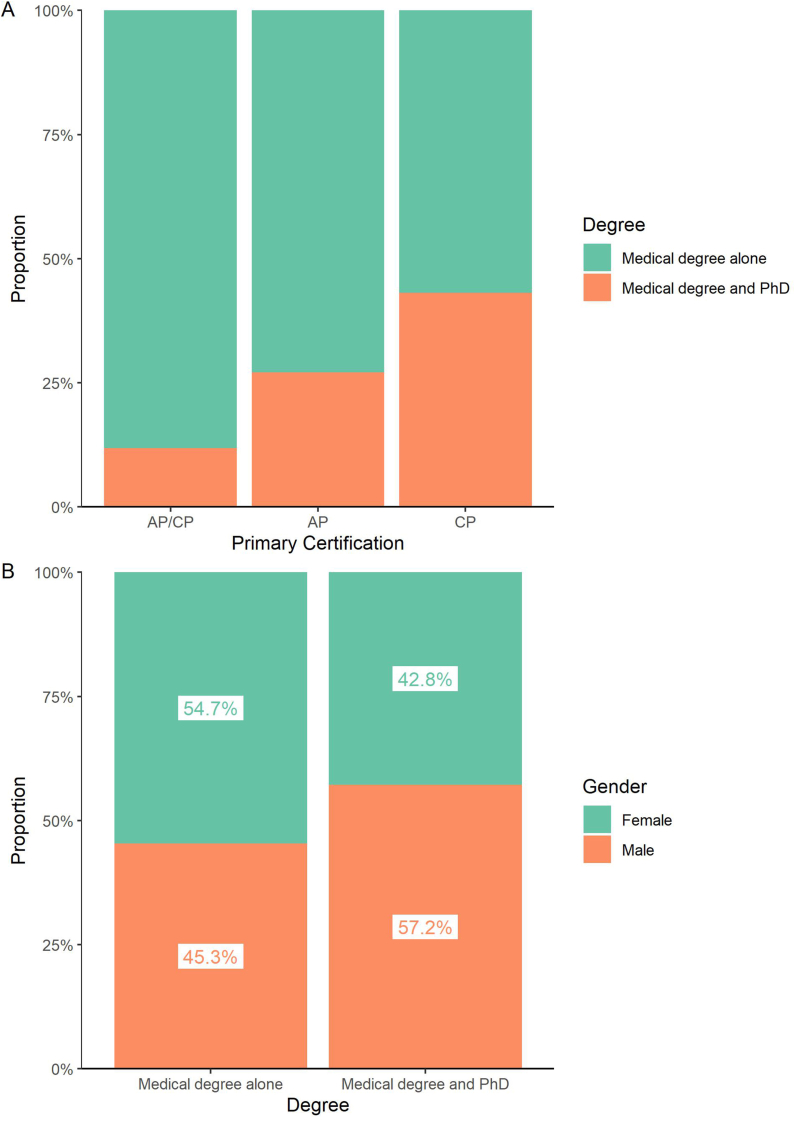
**Association of graduate degrees with gender and primary certification track.** (A) Degrees held by 9578 active pathologists according to primary certification (7630 AP/CP, 1331 AP only, 617 CP only). CP only and, to a lesser extent, AP-only tracks have a larger proportion of PhD holders. (B) Gender representation according to degree held. There is a higher proportion of men among pathologists holding PhDs in addition to their medical degrees. AP, anatomic pathology; CP, clinical pathology.

We asked whether the diversity of pathologists varies according to primary (AP, CP, or combined AP/CP) or secondary specialty. We found a lower proportion of women certified in CP-only (38%) compared to AP-only (52%) or AP/CP (54%; Fig. 4A). Differences in racial diversity were not as pronounced between these tracks (Fig. 4B). Because CP-only training is often sought by graduates of MD/PhD dual-degree programs, which have lower female representation than medical schools overall,^24^ we compared the degrees held by pathologists certified in AP/CP, AP-only, or CP-only (Fig. 5A). We found a higher rate of dual-degree holders (medical degree and PhD) certified in CP-only, and to a lesser extent, AP-only compared to AP/CP. There are more men among the pathologists with MD/PhDs compared to those with other degrees (MD/PhDs 58% male vs MDs: 45% male) (Fig. 5B).

**Fig. 6:**
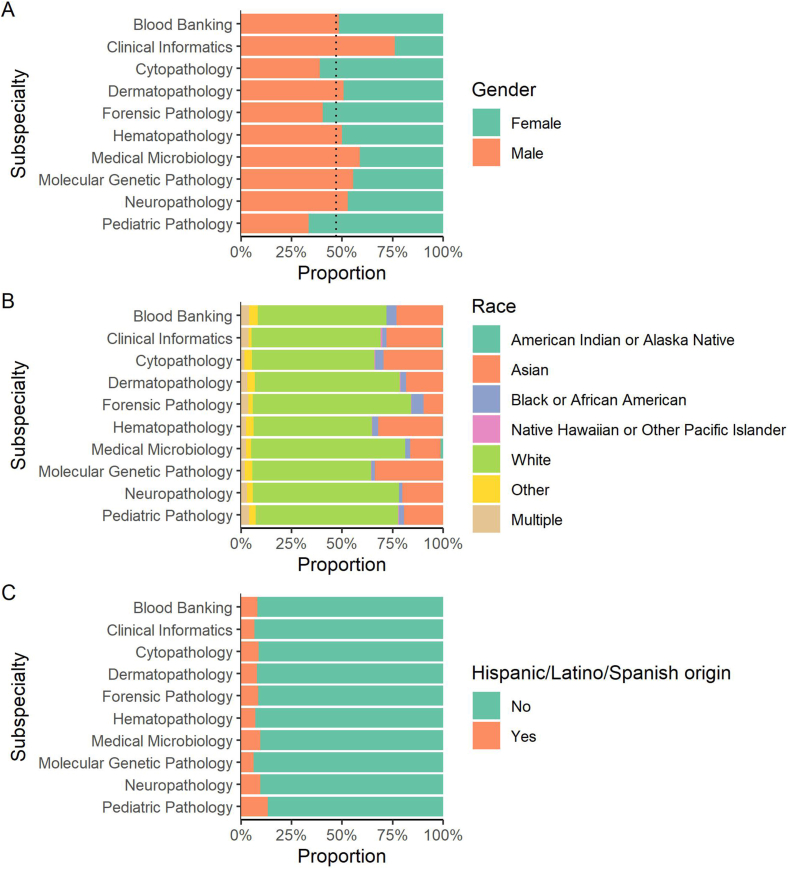
**Gender, race, and ethnicity of active pathologists according to subspecialty certification.** (A) Gender of active pathologists according to subspecialty. The dotted line indicates the overall proportion of active pathologists with continuing certification who are male (47%). While most subspecialties come close to gender parity, there is much less female representation in clinical informatics and more in pediatric pathology. (B) Race of active pathologists according to subspecialty. There is wide variation in Asian representation (ranging from 9% to 35%) and a lesser degree in Black or African American representation (2–6%). (C) Hispanic/Latino/Spanish ethnicity according to subspecialty. Most subspecialties have a comparable proportion of pathologists identifying as Hispanic/Latino/Spanish pathologists, which ranges from 6 to 13%.

Several pathology subspecialties are certified by the American Board of Pathology. We compared gender, race, and ethnicity of active pathologists across these board-certified subspecialties. We found that the diversity of both gender and race varies widely between subspecialties (Fig. 6A and B). Women are severely underrepresented in clinical informatics (25%) and to a lesser extent in medical microbiology (39%). In contrast, other subspecialties (cytopathology, forensic pathology, and pediatric pathology) certify more women than men. Most of the variation in non-White representation between the subspecialties comes from a wide range in Asian representation: Asians represent only 9% of forensic pathologists vs 32% of hematopathologists and 35% of molecular genetic pathologists. Black and African American representation is low in all subspecialties, ranging from 2 to 3% in most subspecialties up to 6% in forensic pathology. There are too few pathologists identifying as American Indian, Alaska Native, or Native Hawaiian or other Pacific Islander for meaningful comparison of these groups between the subspecialties. Individuals with Hispanic/Latino/Spanish ethnicity are similarly underrepresented in most subspecialties (between 6 and 10%), with slightly higher representation (13%) in pediatric pathology but still far below the US Census estimate of 20% of the population (Fig. 6C).^23^

### Demographics of inactive pathologists

**Fig. 7:**
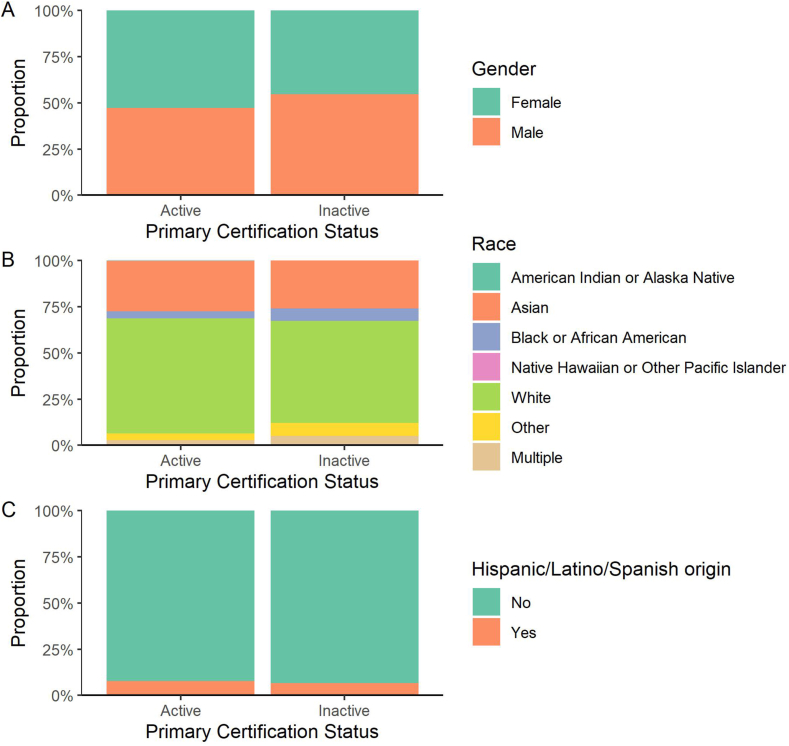
**Gender, race, and ethnicity of pathologists certified in 2006 or later with active vs inactive (expired, surrendered, or revoked) certification.** (A) Comparison of gender among 10,998 pathologists with active certification vs 305 with inactive certification. There is a higher proportion of male pathologists with inactive certification (inactive 56% male, active 47% male). (B) Comparison of race among 10,853 pathologists with active certification vs 59 with inactive certification. (C) Comparison of ethnicity among 10,035 pathologists with active certification vs 61 with inactive certification.

The diversity of the workforce depends on both recruiting and retaining diverse individuals. To examine the retention of US pathologists, we compared the demographics of active pathologists with those whose certification has lapsed, which may reflect departure from the pathology field, still selecting only pathologists certified in 2006 or later (Fig. 7). Fifty-six percent of the pathologists with inactive certification are male, and we do not see evidence that women are disproportionately leaving practice as pathologists. We also do not see obvious differences in race and ethnicity between active and inactive pathologists, but this comparison is limited by the small number of inactive pathologists for whom race and ethnicity data are available (59 for race, 61 for ethnicity).

## Discussion

Here, we comprehensively analyzed the diversity of board-certified pathologists in the US. Consistent with previous data, we observe a substantial improvement in female representation, while several racial and ethnic groups remain underrepresented, meaning their representation among pathologists is lower than their representation in the overall US population: American Indian or Alaska Native pathologists are underrepresented by 7-fold, Black or African American by more than 3-fold, and Hispanic/Latino by more than 2-fold.^5^ Our dataset provides new insight into the wide variation between primary and secondary subspecialties within pathology. Limitations of this study are detailed below and include incomplete historical data (particularly for nonbinary gender identity, race, and ethnicity), lack of granularity in gender, race, and ethnicity categories, and our inability to address other types of identities and experiences related to equity and inclusion.

We observe a substantial increase in female representation from the first board-certified pathologists in the 1930s to the present day, consistent with prior work.^5^ While over 50% of recently certified pathologists are women, reflecting important progress in our field, the overall proportion of women with active certification remains under 50%, as the recent progress has not yet overcome the more widespread exclusion of women in the past. We also found stark differences in female representation across primary and secondary subspecialties, with an especially low proportion of women certified in CP-only (39%) and clinical informatics (25%). These data highlight the need to continue improving female representation in pathology and to ensure that women have equitable opportunities to pursue these more male-dominant subspecialties.

The demographics of dual degree programs may contribute but are insufficient to fully explain the underrepresentation of women in CP-only careers. The data on MD/PhD pathologists are comparable to prior data on MD/PhD program matriculants, which consisted of 37.9% women in 2009 and 46.1% women in 2018.^24^ Factors that affect the lagging female representation in MD/PhD programs, compared to in medical schools as a whole, may include differences in access to research opportunities and mentorship, bias in application review and interviews, and social pressures disproportionately discouraging women from pursuing longer training paths.^25^

Our data are limited in that they only indicate the number of women who are certified, without capturing the full extent of equity and inclusion. Women disproportionately face discrimination, mistreatment, and harassment in the workplace,^26^^,^^27^ and we have not addressed the prevalence of these challenges for women in pathology. Future work is needed to address these issues and to track the extent to which women have access to equitable pay, recognition, respect, and career opportunities.

The inclusion of nonbinary gender as an option in PATHway is an important first step in addressing the wide spectrum of gender identities. In our total dataset, 0.04% of pathologists identified as nonbinary, but the option to specify a nonbinary identity was only recently introduced in 2022, and this is likely an underestimate. Among pathologists certified in 2022 or later, 0.2% indicated that they identify as nonbinary. It is difficult to obtain accurate estimates due to the wide range of identities and the hesitations many lesbian, gay, bisexual, transgender, queer, intersex, and asexual (LGBTQIA) individuals have with disclosing their identity and facing potential bias. One study estimated that there are 1,219,000 nonbinary individuals in the US based on survey data obtained between 2016 and 2018 (approximately 0.4% of the US population).^28^ These estimates suggest that nonbinary individuals continue to be underrepresented among recently certified pathologists. Future work will be important to better evaluate the experience of nonbinary pathologists and address other gender identities and sexual orientations in the LGBTQIA spectrum.

While gender representation in pathology and medicine as a whole has improved, there has been much less progress with respect to racial and ethnic diversity. We note that our historical data regarding race are limited, and since we cannot make meaningful comparisons with data prior to 2006, we focus on the more recent data we have available. We find that among active pathologists certified in 2006 or later, only 0.2% identify as American Indian or Alaska Native (vs 1% of the US population), 3.9% identify as Black or African American (vs 14% of the US population), and 7.9% identify as Hispanic or Latino (vs 20% of the US population). Asian representation is high at 27% of pathologists (vs 6.7% of the US population), but this broad racial category lacks granularity to determine whether specific subgroups are underrepresented.

PATHway provides pathologists with the option to specify an “other” race, which is not offered in the US Census. A small portion of the underrepresentation we observe may be because some individuals indicated a more specific identity in the free text rather than using the broader racial categories used by the US Census, but only 3.7% of pathologists used this option, and we did not frequently find responses specifying an American Indian, Alaska Native, Black, or African American identity. These responses do help to explore the lack of stratification offered by the broad US Census racial categories. We found that the “other” category was most frequently used to specify Middle Eastern/North African, South Asian, and Hispanic/Latin American identities. This corresponds to similar findings from research and community engagement across the overall US population, which led the US Office of Management and Budget to now combine race and ethnicity (allowing for Hispanic or Latine identities) and include a Middle Eastern or North African category for race.^29^ As discussed with respect to gender, these data only reflect the extent to which minority groups are represented in pathology, without addressing the many additional barriers for pathologists from underrepresented groups to be included and treated equitably.

The term intersectionality was coined by Kimberlé Crenshaw to describe the discrimination faced by people with multiple oppressed identities, specifically focusing on violence against Black women.^30^ To explore intersectionality experienced by pathologists, we compared gender representation across different races (Fig. 3). Instead of finding that women of color are excluded, we instead observe much lower male representation among pathologists who identify as Asian or Black or African American. These data correlate with the longstanding underrepresentation of men of color, specifically Black men, among applicants and matriculants to US medical schools.^6^^,^^31^, ^32^, ^33^

Here, we assess only a few of the many levels of diversity, which also include sexual orientation and gender identity (discussed briefly above), neurodiversity, disability status, and nationality, among many others. While these attributes and identities are unavailable in our dataset and beyond the scope of this work, they remain critical aspects of building a workforce that is equitable and best equipped to care for our patients.

While the diversity of pathologists closely follows that of US medical schools, it is important to emphasize that we, as pathologists, share with other medical specialties the responsibility to recruit, retain, and promote diverse physicians. Efforts to improve the diversity, equity, and inclusion (DEI) of physicians must address disparities at every level, including early exposure to medicine, recruitment to medical school and residency programs, and continued support and mentorship of physicians long after completing training.

Efforts to understand and address the sources of these disparities are underway. One important avenue for addressing these disparities is early outreach initiatives to increase exposure to the field of pathology. Pipeline programs have been well established throughout the history of the medical field, and pathology-specific pipeline programs have existed for nearly a century, with the implementation of the first pathology postsophomore fellowship program in 1926.^34^ As these outreach programs have improved overall recruitment into the field of pathology, similar programs are beginning to be used to improve DEI as well. Pathology-specific rotations tailored to students from backgrounds underrepresented in medicine, such as that established by the Johns Hopkins Department of Pathology in 2016, offer a possible model. This fully funded rotation provided a month-long experience for students underrepresented in medicine to rotate through pathology subspecialties, attend research conferences, and meet with faculty. Faculty members are also actively involved in outreach to other institutions, including historically Black medical schools, other underrepresented groups at medical schools, and even high school programs.^35^ Similar programs in other medical subspecialties have shown success in recruitment efforts,^36^^,^^37^ and continuing to develop these outreach initiatives will be an important component of expanding diversity within the field of pathology.

While these types of initiatives can improve the diversity of medical students applying to pathology residency, there are additional disparities that affect which applicants are interviewed at and match into residency programs.^38^ Many of the factors that are used to screen or select residency applicants, such as United States Medical Licensing Examination (USMLE) test scores, clerkship evaluations, letters of recommendation, and Alpha Omega Alpha Honor Medical Society (AOA) membership, can exclude applicants from underrepresented groups without reliably predicting success during residency, particularly when used in isolation.^38^, ^39^, ^40^, ^41^, ^42^, ^43^, ^44^ Holistic review of residency applications and training to reduce implicit bias are therefore important steps to ensure that the match process is equitable for pathology residents.

Ensuring retention and advancement opportunities for pathologists already within the field from underrepresented groups is also crucial. Although we lack data addressing rates of attrition among pathology residents and fellows prior to board certification, data in other specialties including internal medicine, orthopedic surgery, and other surgical subspecialties demonstrate higher rates of attrition among female and POC residents.^45^, ^46^, ^47^ POC resident physicians describe many negative experiences including bias and microaggressions, isolation, and a lack of mentorship.^48^ It will be important for our field to specifically track the experiences and attrition rates of pathology trainees from underrepresented groups. The data from other specialties have led to several recommendations^45^ that can also apply to pathology residency and fellowship programs. Programs can mitigate bias by training faculty and staff in bias and microaggressions, increasing the number and diversity of faculty who perform evaluations, and tracking competency in more objective and measurable ways. Mentorship is essential to support all trainees but especially those who are underrepresented and isolated; programs should provide required, structured, and protected mentorship, with access to mentors from similar backgrounds as the trainees. Our data indicate that pathology trainees do not have equitable access to all subspecialties, and we expect that providing more structured mentorship can help bridge these gaps.

Research regarding job satisfaction, stress, and burnout among laboratory professionals and pathologists has shown a higher prevalence among women, POC, and those with disabilities. Top contributing factors included increased workload, feeling undervalued and undercompensated, experiencing poor management culture, and other work-related stressors such as lack of adequate staffing.^49^ POC participants also experienced higher levels of stress related to being the only person of a specific demographic within a group.^50^ There are also disparities in physician salary, with Black and female physicians receiving lower pay,^51^ and academic promotions, with lower rates of promotion for Black and Hispanic faculty at US medical schools.^52^ Addressing these barriers to retention and advancement will require institutions to standardize salary and promotion criteria, reduce subjectivity and bias in evaluations (similar to the holistic review of residency applications and evaluation of residents and fellows described above), and emphasize the creation of supportive work environments and mentorship programs. Pathologists from underrepresented groups may lack more senior pathologists within their department to provide adequate mentorship. This challenge can be mitigated by peer mentorship programs^53^^,^^54^ and by mentorship spanning across departments or institutions.

These recommendations to improve DEI require considerable work, which often falls on trainees or faculty members from underrepresented backgrounds.^48^ To address this disproportionate burden (the “minority tax”), it is essential that positions such as DEI chairs are created with appropriate time allocated for the work, and that institutions recognize this work when considering career advancement and promotion.

The importance of improving DEI within pathology has also been recognized by several national pathology organizations. The American Society for Clinical Pathology (ASCP) has several dedicated DEI-related initiatives that take a multifactorial approach to improving disparities. These include ambassador programs to improve recruitment, mentoring programs to aid in professional growth, and education programs aimed at training laboratories on implicit bias, cultural competency, and leadership skills.^55^ One product of these initiatives is a report issued in conjunction with the Center for Health Workforce Studies at the University of Washington, which conducted interviews and focus groups with laboratory professionals to delineate opportunities for strengthening workforce recruitment and retention.^56^ Other national organizations, such as the College of American Pathologists (CAP), also have specific committees dedicated to improving DEI within the field, focused on outreach and education efforts.^57^

Despite improvements in physician workforce diversity over the past several decades, much work remains to be done, particularly in light of the recent shifts in national policy aimed at limiting and eliminating DEI initiatives. While there have been many efforts at improving outreach, initiatives to improve retention and professional development are less clearly delineated and formalized. Ultimately, improving diversity within pathology remains an ongoing process that will continue to require a multimodal approach at national, regional, and institutional levels.

## Footnote

This work was presented as a poster at the United States and Canadian Academy of Pathology (USCAP) 2026 Annual Meeting; March 21–26 2026, San Antonio, TX.

## Funding

This research received no specific grant from any funding agency in the public, commercial, or not-for-profit sectors.

## Declaration of competing interest

The authors declare that they have no known competing financial interests or personal relationships that could have appeared to influence the work reported in this paper.

## References

[1] Komaromy M., Grumbach K., Drake M. (1996). The role of black and Hispanic physicians in providing health care for underserved populations. N Engl J Med.

[2] Gomez L.E., Bernet P. (2019). Diversity improves performance and outcomes. J Natl Med Assoc.

[3] Marrast L.M., Zallman L., Woolhandler S., Bor D.H., McCormick D. (2014). Minority physicians' role in the care of underserved patients: diversifying the physician workforce may be key in addressing health disparities. JAMA Intern Med.

[4] Deville C., Hwang W.T., Burgos R., Chapman C.H., Both S., Thomas C.R. (2015). Diversity in graduate medical education in the United States by race, ethnicity, and sex, 2012. JAMA Intern Med.

[5] White M.J., Wyse R.J., Ware A.D., Deville C. (2020). Current and historical trends in diversity by race, ethnicity, and sex within the US pathology physician workforce. Am J Clin Pathol.

[6] U.S. physician workforce data dashboard Association of American medical colleges. https://www.aamc.org/data-reports/report/us-physician-workforce-data-dashboard.

[7] Deville C., Chapman C.H., Burgos R., Hwang W.T., Both S., Thomas C.R. (2014). Diversity by race, Hispanic ethnicity, and sex of the United States medical oncology physician workforce over the past quarter century. J Oncol Pract.

[8] Bae G., Qiu M., Reese E., Nambudiri V., Huang S. (2016). Changes in sex and ethnic diversity in dermatology residents over multiple decades. JAMA Dermatol.

[9] Chapman C.H., Hwang W.T., Both S., Thomas C.R., Deville C. (2014). Current status of diversity by race, Hispanic ethnicity, and sex in diagnostic radiology. Radiology.

[10] Higgins M.C.S.S., Hwang W.T., Richard C. (2016). Underrepresentation of women and minorities in the United States IR academic physician workforce. J Vasc Intervent Radiol.

[11] Chapman C.H., Hwang W.T., Deville C. (2013). Diversity based on race, ethnicity, and sex, of the US radiation oncology physician workforce. Int J Radiat Oncol Biol Phys.

[12] Poon S., Kiridly D., Mutawakkil M. (2019). Current trends in sex, race, and ethnic diversity in orthopaedic surgery residency. J Am Acad Orthop Surg.

[13] Xierali I.M., Nivet M.A., Wilson M.R. (2016). Current and future status of diversity in ophthalmologist workforce. JAMA Ophthalmol.

[14] Lett E., Murdock H.M., Orji W.U., Aysola J., Sebro R. (2019). Trends in racial/ethnic representation among US medical students. JAMA Netw Open.

[15] Cader F.A., Alasnag M., Banerjee S. (2021). Sealing the leaky pipeline: attracting and retaining women in cardiology. Open Heart.

[16] Clarke C.N. (2022). Disparities in creating a diverse surgical oncology physician workforce: just a leaky pipeline?. Surg Oncol Clin.

[17] Daldrup-Link H., Villavasso K., Zhao Q. (2019). How to prevent a leaky pipeline in academic radiology: insights from a faculty survey. J Am Coll Radiol.

[18] Danko D., Cheng A., Losken A. (2021). Gender diversity in plastic surgery: is the pipeline leaky or plugged?. Plast Reconstr Surg.

[19] Sharma N.D., Young K.C., Feld L.D., Rabinowitz L.G. (2024). Patched but still leaky: an update on the pipeline for women in gastroenterology. Dig Dis Sci.

[20] Williams K., Shinkai K. (2022). The leaky pipeline: a narrative review of diversity in dermatology. Cutis.

[21] Hinton A.O., Termini C.M., Spencer E.C. (2020). Patching the leaks: revitalizing and reimagining the STEM pipeline. Cell.

[22] Sarraju A., Ngo S., Rodriguez F. (2023). The leaky pipeline of diverse race and ethnicity representation in academic science and technology training in the United States, 2003–2019. PLoS One.

[23] United States Census Bureau QuickFacts United States census bureau. https://www.census.gov/quickfacts/.

[24] Martinez-Strengel A., Samuels E.A., Cross J. (2022). Trends in U.S. MD-PhD program matriculant diversity by sex and race/ethnicity. Acad Med.

[25] Bannerman C., Guzman N., Kumar R. (2020). Challenges and advice for MD/PhD applicants who are underrepresented in medicine. Mol Biol Cell.

[26] Butkus R., Serchen J., Moyer D.V. (2018). Achieving gender equity in physician compensation and career advancement: a position paper of the American college of physicians. Ann Intern Med.

[27] Chow C.J., Millar M.M., López A.M. (2020). Gender discrimination among academic physicians. Womens Health Rep (New Rochelle).

[28] Wilson B.D.M., Meyer I.H. (2021). https://escholarship.org/uc/item/3kg32337.

[29] Revisions to OMB’s statistical policy directive no. 15: standards for maintaining, collecting, and presenting federal data on race and ethnicity. Fed Regist. March 29, 2024. 2024-06469(89 FR 22182):22182-22186. Accessed 4 September 2025. https://www.federalregister.gov/documents/2024/03/29/2024-06469/revisions-to-ombs-statistical-policy-directive-no-15-standards-for-maintaining-collecting-and.

[30] Crenshaw K. (1991). Mapping the margins: intersectionality, identity politics, and violence against women of color. Stanf Law Rev.

[31] Knox L.D., Seide C.W. (2023). Black men in white coats - barriers black men face in medicine, implications to decreased representation, and potential interventions at the uniformed services university of the health sciences. J Natl Med Assoc.

[32] Laurencin C.T., Murray M. (2017). An American crisis: the lack of Black men in medicine. J Racial Ethn Health Dispar.

[33] (2015). Altering the Course: Black Males in Medicine.

[34] Fenninger L.D. (1958). The Rochester student fellowship program. J Med Educ.

[35] Ware A.D., Murdock T., Voltaggio L. (2019). The “race” toward diversity, inclusion, and equity in pathology: the Johns Hopkins experience. Acad Pathol.

[36] Schukow C., Johnson C., Martinez S., Mckinley K., Campbell K., Ahmed A. (2024). The impact of pathology outreach program (POP) on United States and Canadian high school students. Acad Pathol.

[37] Quintanilla-Arteaga A., McKinley K., Aragao A. (2025). Growing pathology: cultivating interest in pathology within pre-medical students through a novel pathology mini-bootcamp. Acad Pathol.

[38] Ware A.D., Flax L.W., White M.J. (2021). Strategies to enhance diversity, equity, and inclusion in pathology training programs: a comprehensive review of the literature. Arch Pathol Lab Med.

[39] Low D., Pollack S.W., Liao Z.C. (2019). Racial/ethnic disparities in clinical grading in medical school. Teach Learn Med.

[40] Boatright D., Ross D., O'Connor P., Moore E., Nunez-Smith M. (2017). Racial disparities in medical student membership in the Alpha Omega Alpha Honor Society. JAMA Intern Med.

[41] Wagner J.G., Schneberk T., Zobrist M. (2017). What predicts performance? A multicenter study examining the association between resident performance, rank list position, and United States medical licensing examination step 1 scores. J Emerg Med.

[42] Hartman N.D., Lefebvre C.W., Manthey D.E. (2019). A narrative review of the evidence supporting factors used by residency program directors to select applicants for interviews. J Grad Med Educ.

[43] Ross D.A., Boatright D., Nunez-Smith M., Jordan A., Chekroud A., Moore E.Z. (2017). Differences in words used to describe racial and gender groups in medical student performance evaluations. PLoS One.

[44] Gonzalez F., Welsh L., Caicedo J. (2025). Differences in language used to describe racial groups in emergency medicine standardized letter of evaluation. AEM Educ Train.

[45] Reid D., Khadka M. (2025). The urgent need to address residency attrition rates among Black physicians in internal medicine. J Natl Med Assoc.

[46] Haruno L.S., Chen X., Metzger M. (2023). Racial and sex disparities in resident attrition among surgical subspecialties. JAMA Surg.

[47] Haruno L.S., Chen X., Metzger M. (2023). Racial and sex disparities in resident attrition in orthopaedic surgery. JB JS Open Access.

[48] Osseo-Asare A., Balasuriya L., Huot S.J. (2018). Minority resident physicians' views on the role of race/ethnicity in their training experiences in the workplace. JAMA Netw Open.

[49] Garcia E., Kundu I., Kelly M., Soles R., Mulder L., Talmon G.A. (2020). The American society for clinical Pathology's job satisfaction, well-being, and burnout survey of laboratory professionals. Am J Clin Pathol.

[50] Mulder L., Garcia E., Sirintrapun S.J., Kundu I., Soles R. (2024). Examining the role of diversity, equity, and inclusion in mitigating workforce burnout in laboratory medicine. Am J Clin Pathol.

[51] Ly D.P., Seabury S.A., Jena A.B. (2016). Differences in incomes of physicians in the United States by race and sex: observational study. BMJ.

[52] Nunez-Smith M., Ciarleglio M.M., Sandoval-Schaefer T. (2012). Institutional variation in the promotion of racial/ethnic minority faculty at US medical schools. Am J Publ Health.

[53] Fleming G.M., Simmons J.H., Xu M. (2015). A facilitated peer mentoring program for junior faculty to promote professional development and peer networking. Acad Med.

[54] Cree-Green M., Carreau A.M., Davis S.M. (2020). Peer mentoring for professional and personal growth in academic medicine. J Invest Med.

[55] Diversity, Equity & Inclusion American society for clinical pathologists. https://www.ascp.org/about-ASCP/diversity-equity-inclusion.

[56] Garcia E.C., Kundu I., Kelly M.A., Guenther G.A., Skillman S.M., Frogner B.K. (2021). https://familymedicine.uw.edu/chws/wp-content/uploads/sites/5/2021/05/Siemens_Clinical-Laboratory-Workforce_Report_042721.pdf.

[57] Diversity, Equity, Inclusion, and Accessibility Committee (DEIAC) College of American pathologists. https://www.cap.org/member-resources/councils-committees/diversity-equity-inclusion-dei-committee.

